# Algorithm of OMA for large-scale orthology inference

**DOI:** 10.1186/1471-2105-10-220

**Published:** 2009-07-16

**Authors:** Alexander CJ Roth, Gaston H Gonnet, Christophe Dessimoz

**Affiliations:** 1 ETH Zurich, and Swiss Institute of Bioinformatics, 8092 Zurich, Switzerland

## Abstract

Since the publication of our article (Roth, Gonnet, and Dessimoz: BMC Bioinformatics 2008 9: 518), we have noticed several errors, which we correct in the following.

## Correction

We lately identified inadvertent errors in our publication [1]. We regret these errors, and offer our sincere apologizes for the confusion and inconvenience. The corrections are described in detail in what follows.

### Formation of stable pairs

In the main text of section "Formation of stable pairs", the formula for stable pair formation is missing a minus sign after the "greater than" symbol. The sentence should read:

"Formally, a pair of sequences (x, y) from genomes X and Y is considered a stable pair if and only if, for all x_*i *_∈ X, x_*i *_≠ x, and for all y_*j *_∈ Y, y_*j *_≠ y:



and



where *d *is a pairwise maximum likelihood distance estimate and *k*, the tolerance parameter of the standard deviation between the two distances, where ."

### Ortholog clustering

**Figure 1:**
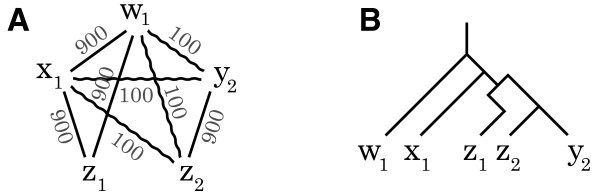
**Corrected Figure Eight – Maximum edge weight cliques for inference of orthologous groups**. **A **An example graph containing one 4-clique, four 3-cliques, and eight 2-cliques is provided. The highest edge scoring partition of the graph is {w_1_, x_1_, z_1_}, {y_2_, z_2_}. **B **A possible evolutionary scenario corresponding to the graph.

In the subsection "Ortholog clustering", the example describing the clustering algorithm suggests that our algorithm could find best global maximum edge weight clique partition. This is incorrect, as our current implementation consists of a k-greedy approximation algorithm which is not guaranteed to find the best global maximum edge weight clique. To avoid any confusion, figure Eight has been updated (Fig. 1 here). The figure caption remains unchanged, but the numbers reported in the main text should now read:

"Figure 1A shows a graph with edges between all vertices except (z_1_, z_2_) and (z_1_, y_2_), which are paralogous relations. The highest scoring partition is {w_1_, x_1_, z_1_}, {y_2_, z_2_}, with the total sum of edge weights of 4·900 = 3600. The score is higher than the highest scoring maximum size clique {w_1_, x_1_, y_2_, z_2_}, {z_1_}, where the sum of the scores is 2·900 + 4·100 = 2200."

### Figure Ten

**Figure 2:**
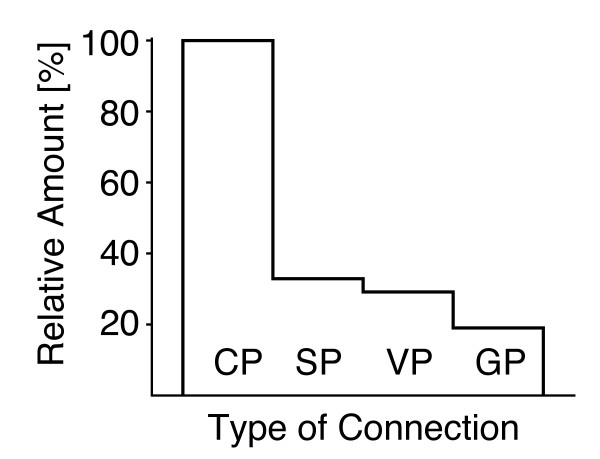
**Corrected Figure Ten – Number of pairs reported after each step**. Each step of the algorithm reduces the number of pairs, and the largest reduction is observed with the formation of stable pairs.

In section "Results and discussion", figure Ten shows the decrease of the relative number of pairs after each step. The y-axis in the figure was scaled incorrectly. A corrected version is provided here (Fig. 2). The caption remains unchanged.

### Bibliography Update

Finally, this correction gives us the opportunity to update a bibliographic reference: since publication of our original manuscript, Reference 33 [2], "in press" then, has been now published. The full reference is provided below.
